# Genetically Predicted Fibroblast Growth Factor 23 and Major Cardiovascular Diseases, Their Risk Factors, Kidney Function, and Longevity: A Two-Sample Mendelian Randomization Study

**DOI:** 10.3389/fgene.2021.699455

**Published:** 2021-07-23

**Authors:** Ying Liang, Shan Luo, C. Mary Schooling, Shiu Lun Au Yeung

**Affiliations:** ^1^LKS Faculty of Medicine, School of Public Health, The University of Hong Kong, Hong Kong, China; ^2^School of Public Health and Health Policy, City University of New York, New York, NY, United States

**Keywords:** FGF23, cardiovascular disease, cardiovascular risk factor, type 2 diabetes mellitus, longevity, kidney disease, Mendelian randomization

## Abstract

**Introduction:**

Fibroblast growth factor 23 (FGF23), a potential biomarker for kidney function, is related to cardiovascular disease (CVD) and diabetes, although it is unclear whether the relation is causal. This study evaluated the associations of genetically predicted FGF23 with major CVDs, their risk factors, kidney function, and longevity using Mendelian randomization (MR).

**Methods:**

This is a two-sample MR study using summary statistics from large genome-wide association studies. Primary outcomes included coronary artery disease (CAD), myocardial infarction, heart failure, and atrial fibrillation. Secondary outcomes included cardiovascular risk factors, kidney function, and longevity. We used four single-nucleotide polymorphisms (SNPs) predicting FGF23, excluding rs2769071 in the *ABO* gene, which likely violates the MR exclusion-restriction assumption. We used inverse-variance weighted (IVW) as the primary statistical method to assess associations of FGF23 with the outcomes. Sensitivity analyses included weighted median (WM) and MR-Egger. We repeated the analyses including all five SNPs. Last, we validated the positive findings from the main analyses in a smaller study, i.e., FinnGen.

**Results:**

Using IVW, genetically predicted higher FGF23 was inversely associated with risk of CAD [odds ratio (OR): 0.69 per logtransformed FGF23 (pg/ml) increase, 95% confidence interval (CI): 0.52–0.91] and type 2 diabetes mellitus (T2DM) (OR: 0.70, 95% CI: 0.52–0.96), but not with the other outcomes. The WM and MR-Egger estimates were directionally consistent.

**Conclusion:**

This study suggests that genetically predicted higher FGF23 may be protective against CAD and T2DM. Future studies should explore the underlying mechanisms related to the potential protective effect of FGF23. FGF23 was unlikely a cause of poorer renal function.

## Introduction

Cardiovascular disease (CVD) is the leading cause of mortality globally (World Health Organization, 2017). According to the Global Burden of Disease Study, an estimated 17.8 million people, around one-third of worldwide deaths, were from CVD in 2017 (Jagannathan et al., 2019). However, causes of CVD remain incompletely understood (Ezzati et al., 2015). Given that kidney diseases are often linked to CVD (Anavekar et al., 2004), recent studies have started to explore the role of kidney-function-related biomarkers, such as fibroblast growth factor 23 (FGF23) in CVD. FGF23 is mainly secreted by osteoblasts and osteocytes and is responsible for phosphate homeostasis (Kocełak et al., 2012). Previous observational studies showed that higher FGF23 was associated with higher risk of major CVDs (Batra et al., 2016), such as hypertension (Fyfe-Johsnon et al., 2016), coronary artery disease (CAD) (Lutsey et al., 2014), myocardial infarction (MI) (Di Giuseppe et al., 2015), atrial fibrillation (AF) (Mathew et al., 2014), and heart failure (HF) (Lutsey et al., 2014). However, a meta-analysis of FGF23 with risk of CVD in 17 general population cohorts suggested no causal relation (Marthi et al., 2018). These discrepancies may indicate the possibility of confounding, reverse causation, and selection bias given that most of these studies were observational.

Mendelian randomization (MR) is a potentially more credible design compared with conventional observational studies given the use of genetic variants randomly allocated at conception and, hence, is more resistant to confounding (Davies et al., 2018; Burgess et al., 2021). Previous MR studies have mainly focused on the associations of genetically predicted FGF23 with bone-related phenotypes, which showed that FGF23 is inversely related to bone mineral density and osteoporosis (Wang et al., 2020; Yokomoto-Umakoshi et al., 2020). Other MR studies also assessed its relation with CAD, stroke, blood pressure, and lipids (Yokomoto-Umakoshi et al., 2020; Zheng et al., 2020), but these studies have not assessed the relation with HF, AF, or type 2 diabetes mellitus (T2DM). Furthermore, since some of these previous studies were conducted in genome-wide association studies (GWAS), which have differences in disease and control definition, analysis model, and study design of the included studies, or may have included potentially invalid instrument such as a highly pleiotropic single-nucleotide polymorphism (SNP) in the *ABO* gene (Folkersen et al., 2020; Wang et al., 2020; Yokomoto-Umakoshi et al., 2020; Zheng et al., 2020). In view of the limited number of FGF23 instruments, such discrepancies can influence the overall assessment of causality for MR studies, as per indicated in previous MR studies such as growth differentiation factor 15 where there are inconsistent findings between CARDIoGRAM and UK Biobank (Au Yeung et al., 2019). To comprehensively evaluate the associations of genetically predicted FGF23 with CVD and its risk factors, we conducted a two-sample MR study using summary statistics from GWAS (Lawlor, 2016). Given that FGF23 is a potential biomarker for kidney function (Robinson-Cohen et al., 2018), we also explored its associations with kidney function for completeness. Finally, as previous studies suggested that FGF23 may be related to longevity from animal studies (Bär et al., 2018), we also assessed its association with longevity.

## Materials and Methods

**FIGURE 1:**
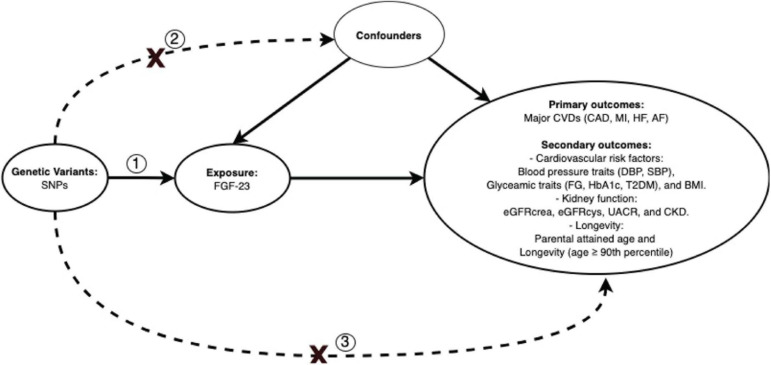
Directed acyclic graph illustrating Mendelian randomization assumptions. Three assumptions should be satisfied: (1) the genetic variants, i.e., SNPs, should predict FGF23; (2) the SNPs should not be associated with potential confounders; (3) the SNPs should only affect the outcomes through affecting FGF23. SNPs, single nucleotide polymorphisms; CAD, coronary artery disease; MI, myocardial infarction; HF, heart failure; AF, atrial fibrillation; SBP, systolic blood pressure; DBP, diastolic blood pressure; FG, fasting glucose; HbA_1c_, glycated hemoglobin; T2DM, type 2 diabetes mellitus; BMI, body mass index; eGFRcrea, estimated glomerular filtration rate from creatinine; eGFRcys, estimated glomerular filtration rate from cystatin C; UACR, urinary albumin-to-creatinine ratio; CKD, chronic kidney disease.

This is an MR study which is based on three assumptions. First, the genetic instruments, i.e., SNPs should predict FGF23. Second, the SNPs should not be associated with potential confounders. Last, the SNPs should only affect the outcome through affecting FGF23 (Figure 1).

### Data Sources

#### Exposure

Genetic predictors of FGF23 were obtained from a meta-analysis of GWAS consisting of seven studies with a total of 16,624 individuals of European ancestry (Robinson-Cohen et al., 2018). The mean age of the study populations ranged from 36.4 to 78.0 years old, and 45.5% (*N* = 7,572) were male. Circulating FGF23 was detected by enzyme-linked immunosorbent assay (ELISA; Kainos Laboratories Inc., Tokyo, Japan) for intact fibroblast growth factor 23 (iFGF23) in 82.5% participants (*N* = 13,716, five cohorts) and by ELISA kit (Immutopics, San Clemente, CA, United States) for C-terminal fragment fibroblast growth factor 23 (cFGF23) in 17.5% participants (*N* = 2,908, two cohorts). The GWAS excluded participants with estimated glomerular filtration rate based on creatinine (eGFRcrea) <30 ml/min per 1.73 m^2^ based on the Chronic Kidney Disease Epidemiology Collaboration (CKD-EPI) equation. FGF23 was log-transformed, and the GWAS was adjusted for age, sex, and the top 10 principal components of ancestry in linear regression (Robinson-Cohen et al., 2018). We considered that SNPs strongly associated with FGF23 (*p*-value < 5 × 10^–8^) and excluded SNPs in high linkage disequilibrium (LD) (*r*^2^ < 0.001). We also excluded the SNP (rs2769071) in the *ABO* gene given that it is known to be highly pleiotropic and, hence, may be an invalid instrument (Supplementary Figure 1; Li and Schooling, 2020), giving four SNPs in the main analysis, and all five in a supplementary analysis.

#### Outcomes

We extracted summary statistics for the outcomes from the relevant GWAS and the IEU GWAS database (Hemani et al., 2018). The primary outcomes included CAD (Nikpay et al., 2015), MI (Nikpay et al., 2015), HF (Shah et al., 2020), and AF (Roselli et al., 2018). We also included cardiovascular risk factors as secondary outcomes, including blood pressure [systolic blood pressure (SBP), diastolic blood pressure (DBP) (Mitchell et al., 2019)], body mass index (BMI) (Yengo et al., 2018), glycaemic traits [fasting glucose (FG) (Lagou et al., 2021), glycated hemoglobin (HbA_1c_) (Wheeler et al., 2017)], and T2DM (restricted to European UK Biobank participants) (Mahajan et al., 2018), but not lipids and stroke because they have been thoroughly investigated in previous studies (Yokomoto-Umakoshi et al., 2020; Zheng et al., 2020). Given that FGF23 is closely related to kidney function (Robinson-Cohen et al., 2018), we also included kidney function as secondary outcomes. These traits included GFRcrea (Wuttke et al., 2019), eGFR based on serum cystatin C (eGFRcys) (Gorski et al., 2017), urinary albumin-to-creatinine ratio (UACR) (Teumer et al., 2019), and chronic kidney disease (CKD) (Wuttke et al., 2019). Last, given that the previous animal studies suggest that reduced FGF23 may decrease lifespan (Bär et al., 2018), we also included parental attained age (i.e., combination of mother and father’s age if alive or at death) in the UK Biobank (Pilling et al., 2017) and longevity (surviving to the 90th percentile age) (Deelen et al., 2019) as secondary outcomes. A proxy SNP in high LD (*r*^2^ ≥ 0.8) was identified if the target SNP was not available for the outcome. LD proxies were obtained using 1,000-genome European phase 3 data. Palindromic SNPs were retained with minor allele frequency <0.3. Details of the data sources are in Table 1.

**TABLE 1:** Information of outcomes included in the study.

**Outcome**	**Abbreviation**	**Unit**	**Consortium**	**PMID**	**Sample size (case / control number)**	**Covariate adjustment**	**Ancestry**
**Major cardiovascular diseases**							
Coronary artery disease (Nikpay et al., 2015)	CAD	log OR	CARDIoGRAMplusC4D 1000 Genomes-based GWAS	26343387	184,305 (*N* case = 60,801, *N* control = 123,504)	study-specific covariates and genomic control	Mixed
Myocardial infarction (Nikpay et al., 2015)	MI	log OR	CARDIoGRAMplusC4D 1000 Genomes-based GWAS	26343387	166,065 (*N* case = 42,561, *N* control = 123,504)	study-specific covariates and genomic control	Mixed
Heart failure (Shah et al., 2020)	HF	log OR	HERMES	31919418	977,323 (*N* case = 47,309, *N* control = 930,014)	Age, sex (except for single-sex studies) and principal components	European
Atrial fibrillation (Roselli et al., 2018)	AF	log OR	2018 AF HRC GWAS	29892015	537,409 (*N* case = 55,114, *N* control = 482,295)	sex, age at first visit, genotyping array and the first ten principal components	European
**Cardiovascular risk factors - Glycaemic traits**							
Fasting glucose (Lagou et al., 2021)	FG	mmol/L	MAGIC	33402679	140,595	age, study site (if applicable), and principal components	European
Glycated hemoglobin (Wheeler et al., 2017)	HbA_1c_	%	MAGIC	28898252	123,665	age, sex, and study-specific covariates	European
Type 2 diabetes mellitus (Mahajan et al., 2018)	T2DM	log OR	DIAMANTE T2D GWAS (restricted to European UK Biobank participants)	29632382	442,817 (*N* case = 19,119, *N* control = 423,698)	study-specific covariates	European
**Cardiovascular risk factors - Blood pressure traits**							
Systolic blood pressure (Mitchell et al., 2019)	SBP	SD	GWAS of UK Biobank	NA	436,419	Genotype array, sex and the first 10 principal components	European
Diastolic blood pressure (Mitchell et al., 2019)	DBP	SD	GWAS of UK Biobank	NA	436,424	Genotype array, sex and the first 10 principal components	European
**Cardiovascular risk factors - BMI**							
Body mass index (Yengo et al., 2018)	BMI	SD	GIANT	30124842	681,275	age, sex, recruitment center, genotyping batches and 10 principal components	European
**Kidney function**							
Creatinine-based estimation of GFR (Wuttke et al., 2019)	eGFRcrea	log ml/min/1.73m^2^	CKDGen	31152163	567,460	Sex, age, study site, genetic principal components, relatedness and other study-specific features	European
Cystatin C–based estimation of GFR (Gorski et al., 2017)	eGFRcys	log ml/min/1.73m^2^	CKDGen	28452372	24,063	Sex, age, study-specific features such as study site or genetic principal components, and relatedness (if family-based studies)	European
Urinary albumin-to-creatinine ratio (Teumer et al., 2019)	UACR	log mg/g	CKDGen	31511532	547,361	Sex, age, study-specific features such as study site or genetic principal components, and relationship of the individuals (if family-based studies)	European
Chronic kidney disease (Wuttke et al., 2019)	CKD	log OR	CKDGen	31152163	480,698 (*N* case = 41,395, *N* control = 439,303)	Sex, age, study site, genetic principal components, relatedness and other study-specific features	European
**Longevity**							
Parental attained age (Pilling et al., 2017)	-	SD	GWAS of UK Biobank	29227965	389,166	offspring age, sex, and genetic principal components 1-5	European
Longevity (age ≥ 90^th^ percentile) (Deelen et al., 2019)	Longevity 90^th^	log OR	CHARGE	31413261	36,745 (*N* case = 11,262, *N* control = 25,483)	clinical site, known family relationships, and/or the first four principal components (if applicable, and genomic control	European

*SNP, single nucleotide polymorphism; CARDIoGRAMplusC4D, Coronary ARtery DIsease Genome wide Replication and Meta-analysis (CARDIoGRAM) plus The Coronary Artery Disease (C4D) Genetics consortium; GWAS, Genome-wide association study; HERMES, The Heart Failure Molecular Epidemiology for Therapeutic Targets; HRC, Haplotype Reference Consortium; MAGIC, Meta-Analyses of Glucose and Insulin-related traits Consortium; DIAMANTE, DIAbetes Meta-ANalysis of Trans-Ethnic association studies; MRC-IEU, Medical Research Council-Integrative Epidemiology Unit; GIANT, Genetic Investigation of ANthropometric Traits; CKDGen, Chronic Kidney Disease Genetics; CHARGE, Cohorts for Health and Aging in genomic Epidemiology; CVD, cardiovascular diseases; CAD, coronary artery disease; MI, myocardial infarction; HF: heart failure; AF, atrial fibrillation; FG, fasting glucose; HbA_1c_, glycated hemoglobin; T2DM, type 2 diabetes mellitus; SBP, systolic blood pressure; DBP, diastolic blood pressure; BMI, body mass index; eGFRcrea, estimated glomerular filtration rate based on creatinine; eGFRcys, estimated glomerular filtration rate based on cystatin C; UACR, urinary albumin-to-creatinine ratio; CKD, chronic kidney disease.*

### Statistical Analyses

We aligned the SNPs on the same allele for exposure and outcome data using allele letter and effect allele frequency (Hartwig et al., 2016). We assessed instrument strength with FGF23 using the *F*-statistic where an *F*-statistic > 10 indicates that a weak instrument bias is unlikely (Bowden et al., 2016; Davies et al., 2018). We calculated the variance (*R*^2^) of FGF23 explained by the instruments using an equation used in previous MR studies (Yarmolinsky et al., 2018; Au Yeung et al., 2021) and used this information to compute the overall *F*-statistic (Yarmolinsky et al., 2018). As our primary statistical method, we assessed the role of FGF23 using inverse-variance weighted (IVW) with multiplicative random effects. IVW assumes all SNPs are valid or have balanced pleiotropy (Bowden and Holmes, 2019). We assessed heterogeneity of the Wald ratios (SNP on outcome divided by on exposure) using Cochran’s *Q*-test where high heterogeneity may indicate the presence of invalid instruments. Details of the statistical analysis methods are in Table 2.

**TABLE 2:** Details of statistical analysis methods used in this Mendelian randomization study.

**Statistical analysis**	**Statistical analysis method**	**Key assumptions**	**Assumption validation**
Primary statistical method	Inverse-variance weighted (IVW)	● Genetic variants satisfy all the three Mendelian randomization assumptions (Figure 1)	● No weak instrument bias: *F*-statistics of each instrument ≥10
		● Hold “No Measurement Error” (NOME) assumption and average horizontal pleiotropic effects of all instruments is zero (balanced pleiotropy)	● No heterogeneity: Cochran’s *Q* is not statistically significant
Sensitivity analysis	MR-Egger	● Require “Instrument Strength Independent of Direct Effect” (InSIDE) assumption	● No horizontal pleiotropic effects: MR-Egger intercept test is not statistically significant. If significant, indicate IVW could be biased
		● All genetic variants can be invalid, as long as InSIDE assumption is fulfilled	
	Weighted median (WM)	● More than 50% of the weight were contributed by valid instruments	

### Sensitivity Analyses

The weighted median (WM) gives valid estimates as long as 50% of the weight is derived from valid SNPs (Bowden et al., 2016). MR-Egger allows for all SNPs to be invalid, if the instrument strength independent of direct effect (InSIDE) assumption is met, i.e., pleiotropic effects of the SNPs are not associated with the strength of SNP on exposure. The MR-Egger intercept (*p*-value < 0.05) indicates the presence of horizontal pleiotropy, i.e., SNPs affect the outcomes through genetic pathways independent of the exposure (Bowden et al., 2015). Directionally consistent findings from different methods may strengthen our findings (Lawlor et al., 2016). We also repeated the analyses including the SNP in the *ABO* gene to assess how the inclusion of this SNP may have affected the results. Last, we used FinnGen (January 14, 2020 release) with a sample size of up to 96,499 to validate any positive findings identified from the main analyses (Supplementary Figure 1).

### Power Calculation

To correct for multiple testing, a Bonferroni corrected *p*-value of 0.0125 (i.e., 0.05/4) was considered as statistical significance for the primary outcomes. At the significance level of 0.0125 and 1.5% variance of FGF23 explained by four SNPs, we calculated statistical power for each primary outcome (Freeman et al., 2013; Burgess, 2014; Supplementary Figure 2).

All analyses were performed using R version 4.0.2 with the R packages (TwoSampleMR, version 0.5.5) (Hemani et al., 2018).

### Ethics Approval

This study only used publicly available data, so no ethical approval is needed.

## Results

We included four SNPs (i.e., rs17216707, rs11741640, rs17479566, and rs9925837), which explained 1.5% of the variance of FGF23 in the main analysis (Supplementary Figure 1). The overall *F*-statistic of the four SNPs was 63, and *F*-statistic of each SNP was larger than 10, indicating that a weak instrument bias is unlikely. Details of the instruments can be found in Supplementary Table 1 and Table 1.

**FIGURE 2:**
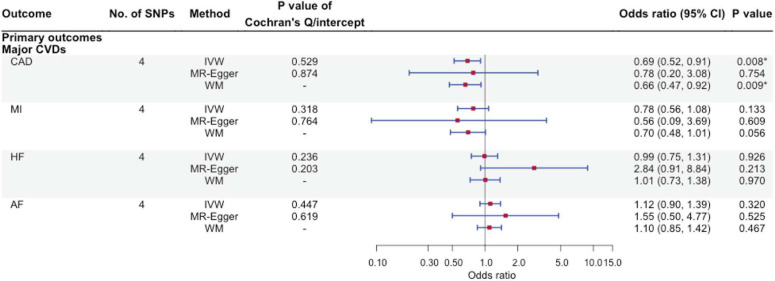
The associations of genetically predicted FGF23 and major cardiovascular diseases using Mendelian randomization. No. of SNPs, number of single nucleotide polymorphisms; IVW, inverse-variance weighted; WM, weighted median; CVD, cardiovascular disease; CAD, coronary artery disease; MI, myocardial infarction; HF, heart failure; AF, atrial fibrillation. ^∗^Bonferroni-corrected *P*-value < 0.0125.

Using IVW, higher FGF23 was associated with lower CAD risk [odds ratio (OR): 0.69 per natural log transformed FGF23 increase, 95% confidence interval (CI): 0.52–0.91], with directionally consistent findings from WM and MR-Egger. Similar findings were observed for MI. However, FGF23 was not associated with AF and HF although with wide 95% CIs. There was no evidence for heterogeneity based on the Cochran’s *Q*-test or MR-Egger intercept (Figure 2).

**FIGURE 3:**
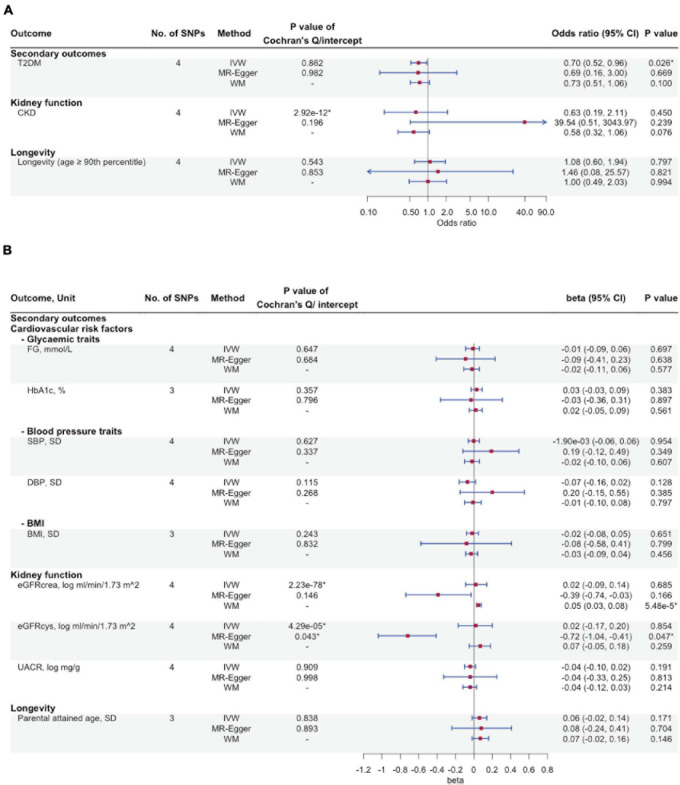
The associations of genetically predicted FGF23 and cardiovascular risk factors, kidney function, and longevity using Mendelian randomization. **(A)** Type 2 diabetes mellitus, chronic kidney disease, and longevity; **(B)** Glycaemic traits, blood pressure traits, BMI, kidney function and longevity. No. of SNPs, number of single nucleotide polymorphisms; IVW, inverse-variance weighted; WM, weighted median; T2DM, type 2 diabetes mellitus; CKD, chronic kidney disease; FG, fasting glucose; HbA_1c_, glycated hemoglobin; SBP, systolic blood pressure; DBP, diastolic blood pressure; BMI, body mass index; eGFRcrea, estimated glomerular filtration rate from creatinine; eGFRcys, estimated glomerular filtration rate from cystatin C; UACR, urinary albumin-to-creatinine ratio. ^∗^*P*-value < 0.05.

Figures 3A,B show the association of FGF23 with the secondary outcomes, including cardiovascular risk factors, kidney function, and longevity. We found an inverse association of FGF23 with T2DM risk (IVW OR: 0.70 per natural log transformed FGF23 increase, 95% CI: 0.52–0.96), with directionally consistent findings from WM and MR-Egger. However, FGF23 was not associated with CKD or with glycemic traits, blood pressure, BMI, kidney function, or longevity. Supplementary Table 2 gives associations of the SNPs with all outcomes.

We also assessed the associations of FGF23 with CAD and T2DM using a separate study (FinnGen study) as a verification (Supplementary Table 3), where we found directionally consistent findings using IVW and WM (Supplementary Table 4). However, the MR-Egger estimate was in the opposite direction with wide 95% CI.

In sensitivity analysis where we included rs2769071, a variant in the *ABO* gene, associations with CAD, MI, and T2DM were attenuated, while FGF23 remained unrelated to the other outcomes. However, the Cochran’s *Q*-statistics indicated heterogeneity for many analyses, suggesting including rs2769071 may invalidate the analyses, in particular, for IVW (Supplementary Figures 3, 4A,B and Supplementary Tables 2, 5).

## Discussions

In this MR study, which included a comprehensive range of cardiovascular outcomes, their risk factors, kidney function, and longevity, inconsistent with previous observational studies, we found that genetically predicted FGF23 was inversely associated with CAD, MI, and T2DM but not with other cardiovascular outcomes or risk factors. We also found no strong evidence that FGF23 is related to kidney function. Last, we did not find evidence for an effect of genetically predicted FGF23 on longevity. Our study adds by clarifying, using genetic evidence, associations of FGF23 with CVD, T2DM, and kidney function, unbiased by confounding, as well as explaining the discrepant results in previous studies.

Previous observational studies found that FGF23 is positively associated with risk of CVD (Lutsey et al., 2014; Mathew et al., 2014; Di Giuseppe et al., 2015). However, these findings were not consistently observed in studies in animals and humans (Takashi et al., 2017; Liu et al., 2018; Pastor-Arroyo et al., 2018). Inconsistent findings may be due to confounding, for example, by socioeconomic position and lifestyle factors. Using a design, which is more resistant to confounding, we found an inverse association of FGF23 with CAD and MI. These findings are consistent with animal studies where lack of FGF23 increased the risk of age-related diseases (Bär et al., 2018).

The mechanisms underlying the effects of FGF23 remain unclear. FGF23 may decrease calcium reabsorption, increase phosphate excretion, and reduce vitamin D production (Liu and Quarles, 2007). Given that calcium is increasingly recognised as a cause of CAD (Xu et al., 2017), this may be one of the possible pathways by which FGF23 reduces the risk of CAD. Another possible pathway is coagulation, where the FGF23 increasing allele G of rs11741640 is strongly associated with prolonged activated partial thromboplastin time (*p*-value: 4.96 × 10^–93^) in Biobank Japan (Hemani et al., 2018; Kanai et al., 2018). Alternatively, the protective effect on CAD could be mediated via reduced risk of T2DM (Ahmad et al., 2015). Regarding the inverse association of FGF23 in T2DM, although FGF23 may reduce vitamin D (Gutiérrez, 2010), a recent MR study showed no strong evidence for an association of FGF23 in vitamin D levels (Wang et al., 2020). Taking into account the potential protective effect of vitamin D on T2DM risk (Yuan et al., 2019), any potential protective effect of FGF23 on T2DM is unlikely via vitamin D pathways. The inconsistent associations with glycemic traits, such as HbA_1c_ and FG, also warrant future investigations. Better understanding of the role of FGF23 and closely related membrane-bound protein (e.g., klotho) (Razzaque, 2009) may also shed light on the mechanisms of medications known to have pleiotropic effects, such as metformin, whose pharmacological target, AMPK (Luo et al., 2020b), is a regulator of FGF23 production (Glosse et al., 2018).

Fibroblast growth factor 23 was not clearly associated with kidney function or CKD (Figures 2, 3A,B and Supplementary Figures 3, 4A,B), although associations have been seen in some (Larsson et al., 2003; Fliser et al., 2007) but not all studies (Levin et al., 2014; Alderson et al., 2016). These inconsistent findings may be due to selection bias as some of these studies were conducted in CKD patients. Alternatively, FGF23 could be a consequence of CKD instead of a cause (Fauconnier et al., 2019). The lack of association of FGF23 with kidney-related outcomes partly supports this argument. Whether CKD impacts FGF23 (i.e., reverse causation) can be explored using a bi-directional MR design in the future, when suitable studies are available such as genetic summary statistics of FGF23 becomes available.

Findings differed with or without inclusion of the SNP from the *ABO* gene (Figures 2, 3A,B and Supplementary Figures 3, 4A,B), which may help explain discrepancies across different MR studies (Folkersen et al., 2020). Other reasons accounting for discrepancies may include the choice of the outcome GWAS. For example, the inverse association of FGF23 with CAD was only observed in this MR but not a previous study, which included UK Biobank (Yokomoto-Umakoshi et al., 2020). Given that only a few SNPs were available for FGF23, the magnitude and direction of genetic associations can be influenced more easily due to differences in disease definition used in the respective GWAS, the inclusion of UK Biobank, and different analytic models, which requires further investigations.

Despite using a study design less susceptible to confounding than typical observational studies, there were some limitations. First, MR has stringent assumptions. We chose instruments for FGF23 from the most recent GWAS; previous studies suggested that FGF23 is predictive of bone mineral density, which is a known effect of FGF23 (Wang et al., 2020). Given that genetic variants were randomly allocated at conception, these instruments are unlikely to be confounded. It is more difficult to assess violation of the exclusion restriction assumption (i.e., instruments affect the outcomes other than via affecting the exposure) given the limited statistical power of sensitivity analyses with a small number of SNPs. This is particularly problematic for MR-Egger and could have explained the differences with other sensitivity analyses in the presence of outliers. Nevertheless, given that *ABO* gene is highly pleiotropic, we removed this SNP in the main analysis to reduce the likelihood of violation of the exclusion restriction assumption. Heterogeneity was higher when this SNP was included (Supplementary Figures 3, 4A,B). However, we could not rule out the possibility of horizontal pleiotropy in other SNPs. For example, rs11741640 was associated with some hematological traits (e.g., hemoglobin), and our previous MR studies suggest that hemoglobin may play a role in venous thromboembolism but not CAD (Zhong et al., 2016; Luo et al., 2020a). As such, replication of our findings using larger GWAS of FGF23 with more genetic instruments, including rare variants, which may have larger effect sizes, is warranted. Second, MR studies are also subject to selection bias, which may explain the null findings concerning HF and AF, which usually occurred at older ages (Schooling et al., 2021b), although FGF23 was not associated with longevity making this explanation less likely. Third, some of the studies in the FGF23 GWAS were also included in the outcome GWAS, although the proportion overlap was generally very small given the small sample size of the FGF23, apart from the eGFRcys GWAS (45%) (Supplementary Table 6). This implies the presence of weak instrument bias, the MR estimates would be biased toward null, although the *F*-statistics indicated low evidence for weak instrument bias (Burgess et al., 2016). Fourth, it is also increasingly recognised that covariable adjustment, either in the form of analysis or study design, may bias the MR estimates, which is one limitation regarding the use of summary statistics (Hartwig et al., 2021; Schooling et al., in press). Furthermore, it is possible that the effect of FGF23 on cardiovascular outcomes was sex specific, as evident in its relation with bone mineral density (Manolagas et al., 2013; Kodrič et al., 2019). However, we were unable to conduct sex-specific analyses given the lack of comprehensive summary statistics. Last, we were unable to conduct a bi-directional MR study to assess whether poorer kidney function increased FGF23 due to lack of relevant genetic summary statistics.

In conclusion, this MR study suggests that genetically predicted higher FGF23 may be protective against CAD and T2DM. Future studies should explore the mechanisms underpinning such relations, which may help explain the discrepant results concerning the role of FGF23 in CVD, T2DM, and kidney diseases across different studies, and further elucidate the role of FGF23 in these diseases.

## Data Availability Statement

The original contributions presented in the study are included in the article/Supplementary Material, further inquiries can be directed to the corresponding author/s.

## Ethics Statement

This study only used publicly available summary statistics from relevant genome-wide association studies (GWAS) and UK Biobank and hence no ethics approval was required. Respective ethics approval have been obtained by the GWAS and the UK Biobank investigators.

## Author Contributions

SLAY and YL designed the study. YL wrote the analysis plan and interpreted the results, with feedback from SL and SLAY. YL wrote the first draft of the manuscript with critical feedback and revisions from SL, SLAY, and CMS. SLAY was the guarantor of the work. All authors contributed to the article and approved the submitted version.

## Conflict of Interest

The authors declare that the research was conducted in the absence of any commercial or financial relationships that could be construed as a potential conflict of interest.

## Publisher’s Note

All claims expressed in this article are solely those of the authors and do not necessarily represent those of their affiliated organizations, or those of the publisher, the editors and the reviewers. Any product that may be evaluated in this article, or claim that may be made by its manufacturer, is not guaranteed or endorsed by the publisher.

## Supplementary Material

Supplementary Figure 1 Study design of this Mendelian randomization study of genetically predicted FGF23 and cardiovascular diseases, their risk factors, kidney function, and longevity.

Supplementary Figure 2 Power estimates for this Mendelian randomization study of FGF23 in major cardiovascular outcomes.

Supplementary Figure 3 The associations of genetically predicted FGF23 and major cardiovascular diseases using Mendelian randomization, including the rs2769071 in *ABO* gene.

Supplementary Figure 4 The associations of genetically predicted FGF23 on cardiovascular risk factors, kidney function, and longevity using Mendelian randomization, including the rs2769071 in *ABO* gene.

Supplementary Table 1 Genetic predictors of log-transformed FGF23.

Supplementary Table 2 Genetic associations of major CVDs, their risk factors, kidney function, and longevity.

Supplementary Table 3 Genetic associations of CVDs and T2DM in FinnGen study.

Supplementary Table 4 The associations of genetically predicted FGF23 and CAD and T2DM in FinnGen study using Mendelian randomization.

Supplementary Table 5 Genetic association of rs2769071 in *ABO* gene with major CVD, their risk factors, kidney function, and longevity.

Supplementary Table 6 Participant overlap between the FGF23 genome wide association studies (GWAS) and the outcome GWAS.
