# Corrigendum: Genetically Predicted Fibroblast Growth Factor 23 and Major Cardiovascular Diseases, Their Risk Factors, Kidney Function, and Longevity: A Two-Sample Mendelian Randomization Study

**DOI:** 10.3389/fgene.2021.794246

**Published:** 2021-11-11

**Authors:** Ying Liang, Shan Luo, C. Mary Schooling, Shiu Lun Au Yeung

**Affiliations:** ^1^ LKS Faculty of Medicine, School of Public Health, University of Hong Kong, Pokfulam, Hong Kong SAR, China; ^2^ School of Public Health and Health Policy, City University of New York, New York, NY, United States

**Keywords:** FGF23, cardiovascular disease, cardiovascular risk factor, type 2 diabetes mellitus, longevity, kidney disease, Mendelian randomization

In the original article, there was an error where the description of Type 2 Diabetes Miletus (T2DM) under **Data Sources, Outcomes** was not clear. In this study, the T2DM data “restricted to European UK Biobank participants” was used.

A correction has been made to **Data Sources, Outcomes**:

“We also included cardiovascular risk factors as secondary outcomes, including blood pressure [systolic blood pressure (SBP), diastolic blood pressure (DBP) (Mitchell et al., 2019)], body mass index (BMI) (Yengo et al., 2018), glycaemic traits [fasting glucose (FG) (Lagou et al., 2021), glycated hemoglobin (HbA_1c_) (Wheeler et al., 2017)], and T2DM (restricted to European UK Biobank participants) (Mahajan et al., 2018),”

In addition, there were mistakes in Table 1, Supplementary Table S6, and Supplementary Figure S1 as published when describing the genetic data used for T2DM. The sample size number of T2DM (restricted to European UK Biobank participants) including case and control number was incorrect. The corrected Table 1, Supplementary Table S6, and Supplementary Figure S1 appear below.

**TABLE 1:** Information of outcomes included in the study.

Outcome	Abbreviation	Unit	Consortium	PMID	Sample size (case/control number)	Covariate adjustment	Ancestry
Major cardiovascular diseases							
Coronary artery disease (Nikpay et al., 2015)	CAD	log OR	CARDIoGRAMplusC4D 1000 Genomes-based GWAS	26343387	184,305 (*N* case = 60,801, *N* control = 123,504)	Study-specific covariates and genomic control	Mixed
Myocardial infarction (Nikpay et al., 2015)	MI	log OR	CARDIoGRAMplusC4D 1000 Genomes-based GWAS	26343387	166,065 (*N* case = 42,561, *N* control = 123,504)	Study-specific covariates and genomic control	Mixed
Heart failure (Shah et al., 2020)	HF	log OR	HERMES	31919418	977,323 (*N* case = 47,309, *N* control = 930,014)	Age, sex (except for single-sex studies) and principal components	European
Atrial fibrillation (Roselli et al., 2018)	AF	log OR	2018 AF HRC GWAS	29892015	537,409 (*N* case = 55,114, *N* control = 482,295)	Sex, age at first visit, genotyping array and the first ten principal components	European
Cardiovascular risk factors—glycaemic traits							
Fasting glucose (Lagou et al., 2021)	FG	mmol/L	MAGIC	33402679	140,595	Gge, study site (if applicable), and principal components	European
Glycated hemoglobin (Wheeler et al., 2017)	HbA_1c_	%	MAGIC	28898252	123,665	Age, sex, and study-specific covariates	European
Type 2 diabetes mellitus (Mahajan et al., 2018)	T2DM	log OR	DIAMANTE T2D GWAS (restricted to European UK Biobank participants)	29632382	442,817 (*N* case = 19,119, *N* control = 423,698)	Study-specific covariates	European
Cardiovascular risk factors—blood pressure traits							
Systolic blood pressure (Mitchell et al., 2019)	SBP	SD	GWAS of UK Biobank	NA	436,419	Genotype array, sex and the first 10 principal components	European
Diastolic blood pressure (Mitchell et al., 2019)	DBP	SD	GWAS of UK Biobank	NA	436,424	Genotype array, sex and the first 10 principal components	European
Cardiovascular risk factors—BMI							
Body mass index (Yengo et al., 2018)	BMI	SD	GIANT	30124842	681,275	Age, sex, recruitment centre, genotyping batches and 10 principal components	European
Kidney function							
Creatinine-based estimation of GFR (Wuttke et al., 2019)	eGFRcrea	log ml/min/1.73 m^2^	CKDGen	31152163	567,460	Sex, age, study site, genetic principal components, relatedness and other study-specific features	European
Cystatin C–based estimation of GFR (Gorski et al., 2017)	eGFRcys	log ml/min/1.73 m^2^	CKDGen	28452372	24,063	Sex, age, study-specific features such as study site or genetic principal components, and relatedness (if family-based studies)	European
Urinary albumin-to-creatinine ratio (Teumer et al., 2019)	UACR	log mg/g	CKDGen	31511532	547,361	Sex, age, study-specific features such as study site or genetic principal components, and relationship of the individuals (if family-based studies)	European
Chronic kidney disease (Wuttke et al., 2019)	CKD	log OR	CKDGen	31152163	480,698 (*N* case = 41,395, *N* control = 439,303)	Sex, age, study site, genetic principal components, relatedness and other study-specific features	European
Longevity							
Parental attained age (Pilling et al., 2017)	—	SD	GWAS of UK Biobank	29227965	389,166	Offspring age, sex, and genetic principal components 1–5	European
Longevity (age ≥ 90th percentile) (Deelen et al., 2019)	Longevity 90th	log OR	CHARGE	31413261	36,745 (*N* case = 11,262, *N* control = 25,483)	Clinical site, known family relationships, and/or the first four principal components (if applicable, and genomic control	European

SNP, single nucleotide polymorphism; CARDIoGRAMplusC4D, Coronary ARtery DIsease Genome wide Replication and Meta-analysis (CARDIoGRAM) plus The Coronary Artery Disease (C4D) Genetics consortium; GWAS, genome-wide association study; HERMES, The HEart failure Molecular Epidemiology for Therapeutic Targets; HRC, Haplotype Reference Consortium; MAGIC, Meta-Analyses of Glucose and Insulin-related traits Consortium; DIAMANTE, DIAbetes Meta-ANalysis of Trans-Ethnic association studies; MRC-IEU, Medical Research Council-Integrative Epidemiology Unit; GIANT, Genetic Investigation of ANthropometric Traits; CKDGen, Chronic Kidney Disease Genetics; CHARGE, Cohorts for Health and Aging in genomic Epidemiology; CVD, cardiovascular diseases; CAD, coronary artery disease; MI, myocardial infarction; HF, heart failure; AF, atrial fibrillation; FG, fasting glucose; HbA_1c_, glycated hemoglobin; T2DM, type 2 diabetes mellitus; SBP, systolic blood pressure; DBP, diastolic blood pressure; BMI, body mass index; eGFRcrea, estimated glomerular filtration rate based on creatinine; eGFRcys, estimated glomerular filtration rate based on cystatin C; UACR, urinary albumin-to-creatinine ratio; CKD, chronic kidney disease.

The authors apologize for these errors and state that this does not change the scientific conclusions of the article in any way. The original article has been updated.

## Supplementary Material

Supplementary Figure S1 Study design of this Mendelian randomization study of genetically predicted FGF23 and cardiovascular diseases, their risk factors, kidney function and longevity. SNP, single nucleotide polymorphism; LD, linkage disequilibrium; CARDIoGRAMplusC4D, Coronary ARtery DIsease Genome wide Replication and Meta-analysis (CARDIoGRAM) plus The Coronary Artery Disease (C4D) Genetics consortium; GWAS, genome-wide association study; HERMES, The Heart Failure Molecular Epidemiology for Therapeutic Targets; HRC, Haplotype Reference Consortium; MAGIC, Meta-Analyses of Glucose and Insulin-related traits Consortium; DIAMANTE, DIAbetes Meta-ANalysis of Trans-Ethnic association studies; MRC-IEU, Medical Research Council-Integrative Epidemiology Unit; GIANT, Genetic Investigation of ANthropometric Traits; CKDGen, Chronic Kidney Disease Genetics; CHARGE, Cohorts for Health and Aging in genomic Epidemiology; CVD, cardiovascular diseases; CAD, coronary artery disease; MI, myocardial infarction; HF, heart failure; AF, atrial fibrillation; FG, fasting glucose; HbA_1c_, glycated hemoglobin; T2DM, type 2 diabetes mellitus; SBP, systolic blood pressure; DBP, diastolic blood pressure; BMI, body mass index; eGFRcrea, estimated glomerular filtration rate based on creatinine; eGFRcys, estimated glomerular filtration rate based on cystatin C; UACR, urinary albumin-to-creatinine ratio; CKD, chronic kidney disease.

Supplementary Table S6 Participant overlap between the FGF23 genome wide association studies (GWAS) and the outcome GWAS.
