# Supplementary material for: Genetically Predicted Fibroblast Growth Factor 23 and Major Cardiovascular Diseases, Their Risk Factors, Kidney Function, and Longevity: A Two-Sample Mendelian Randomization Study
Source: Front Genet. 2021 Jul 23;12:699455. doi: 10.3389/fgene.2021.699455 (PMC8343174; doi:10.3389/fgene.2021.699455)
Supplement: Supplementary Figure 1 — Study design of this Mendelian randomization study of genetically predicted FGF23 and cardiovascular diseases, their risk factors, kidney function, and longevity. [file Data_Sheet_1.pdf]

Supplementary Material

Supplementary Table 1. Genetic predictors of log-transformed FGF23.

SNP	Nearest Gene[a]	Chr: position	EA	OA	EAF[b]	Beta[c]	SE[c]	P value[c]	R^2	F-statistic	Main analysis	Sensitivity analysis
rs17216707	*CYP24A1*	20:52732362	T	C	0.8	0.054	0.005	3.0×10^{-24}	0.007	117.5	✓	✓
rs2769071	*ABO*	9:136145974	G	A	0.37	0.037	0.005	6.1×10^{-17}	0.0033	54.8		✓
rs11741640	*RGS14*	5:176792743	G	A	0.73	0.039	0.005	1.6×10^{-16}	0.0036	60.8	✓	✓
rs17479566	*LINC01506*	9:71198014	T	C	0.22	0.031	0.005	2.0×10^{-09}	0.0024	39.2	✓	✓
rs9925837	*LINC01229*	16:79927303	G	A	0.13	0.035	0.006	5.1×10^{-09}	0.002	34.0	✓	✓

SNP, single nucleotide polymorphism; Chr, chromosome; EA, effect allele; OA, other allele; EAF, effect allele frequency; SE, standard error.
[a] Nearest gene by physical distance to the lead SNP
[b] Allele frequency data from 1000 Genomes Phase 1 genotype data.
[c] Based on a fixed-effects inverse-variance weighted meta-analysis of log-transformed FGF23 association by adjusting age, sex, and the top 10 principal components of ancestry.

Supplementary Table 2. Genetic associations of major CVDs, their risk factors, kidney function and longevity.

SNP	EA	OA	FGF23				Outcome	Outcome			
			Beta	SE	EAF	P value		Beta	SE	EAF	P value
rs11741640	G	A	0.039	0.005	0.730	1.59E-16	CAD	-0.022	0.010	0.738	0.039
rs17216707	T	C	0.054	0.005	0.800	3.00E-24	CAD	-0.018	0.012	0.803	0.130
rs17479566	T	C	0.031	0.005	0.221	2.01E-09	CAD	-0.018	0.011	0.232	0.100
rs9925837	G	A	0.035	0.006	0.130	5.11E-09	CAD	0.002	0.013	0.156	0.870
rs11741640	G	A	0.039	0.005	0.730	1.59E-16	MI	-0.015	0.012	0.747	0.199
rs17216707	T	C	0.054	0.005	0.800	3.00E-24	MI	-0.018	0.013	0.810	0.161
rs17479566	T	C	0.031	0.005	0.221	2.01E-09	MI	-0.014	0.012	0.221	0.249
rs9925837	G	A	0.035	0.006	0.130	5.11E-09	MI	0.016	0.014	0.152	0.269
rs11741640	G	A	0.039	0.005	0.730	1.59E-16	HF	-0.007	0.009	0.710	0.430
rs17216707	T	C	0.054	0.005	0.800	3.00E-24	HF	0.013	0.010	0.807	0.192
rs17479566	T	C	0.031	0.005	0.221	2.01E-09	HF	-0.013	0.010	0.216	0.161
rs9925837	G	A	0.035	0.006	0.130	5.11E-09	HF	7.00E-04	0.011	0.153	0.947
rs11741640	G	A	0.039	0.005	0.730	1.59E-16	AF	-0.001	0.008	NA	0.865
rs17216707	T	C	0.054	0.005	0.800	3.00E-24	AF	0.011	0.010	NA	0.268
rs17479566	T	C	0.031	0.005	0.221	2.01E-09	AF	-0.004	0.009	NA	0.629
rs9925837	G	A	0.035	0.006	0.130	5.11E-09	AF	0.014	0.010	NA	0.143
rs11741640	G	A	0.039	0.005	0.730	1.59E-16	FG	0.002	0.005	NA	0.623
rs17216707	T	C	0.054	0.005	0.800	3.00E-24	FG	-0.002	0.003	NA	0.433
rs17479566	T	C	0.031	0.005	0.221	2.01E-09	FG	-0.002	0.003	NA	0.539
rs9925837	G	A	0.035	0.006	0.130	5.11E-09	FG	0.002	0.003	NA	0.450
rs17216707	T	C	0.054	0.005	0.800	3.00E-24	HbA_{1c}	0.001	0.002	0.788	0.581
rs17479566	T	C	0.031	0.005	0.221	2.01E-09	HbA_{1c}	0.003	0.002	0.196	0.122
rs9925837	G	A	0.035	0.006	0.130	5.11E-09	HbA_{1c}	-0.001	0.002	0.173	0.629
rs11741640	G	A	0.039	0.005	0.730	1.59E-16	T2DM	-0.022	0.012	0.700	0.054
rs17216707	T	C	0.054	0.005	0.800	3.00E-24	T2DM	-0.017	0.013	0.800	0.210
rs17479566	T	C	0.031	0.005	0.221	2.01E-09	T2DM	-0.009	0.013	0.200	0.480

SNP	EA	OA	FGF23				Outcome	Outcome			
			Beta	SE	EAF	P value		Beta	SE	EAF	P value
rs9925837	G	A	0.035	0.006	0.130	5.11E-09	T2DM	-0.006	0.014	0.100	0.690
rs11741640	G	A	0.039	0.005	0.730	1.59E-16	SBP	-0.001	0.002	0.736	0.570
rs17216707	T	C	0.054	0.005	0.800	3.00E-24	SBP	0.003	0.003	0.810	0.330
rs17479566	T	C	0.031	0.005	0.221	2.01E-09	SBP	-0.001	0.003	0.219	0.590
rs9925837	G	A	0.035	0.006	0.130	5.11E-09	SBP	-0.001	0.003	0.150	0.660
rs11741640	G	A	0.039	0.005	0.730	1.59E-16	DBP	-0.006	0.002	0.736	0.015
rs17216707	T	C	0.054	0.005	0.800	3.00E-24	DBP	1.69E-04	0.003	0.810	0.950
rs17479566	T	C	0.031	0.005	0.221	2.01E-09	DBP	-0.005	0.003	0.219	0.033
rs9925837	G	A	0.035	0.006	0.130	5.11E-09	DBP	-4.31E-04	0.003	0.150	0.880
rs11741640	G	A	0.039	0.005	0.730	1.59E-16	BMI	-0.002	0.002	0.716	0.210
rs17216707	T	C	0.054	0.005	0.800	3.00E-24	BMI	-9.00E-04	0.002	0.808	0.680
rs9925837	G	A	0.035	0.006	0.130	5.11E-09	BMI	0.003	0.002	0.157	0.240
rs11741640	G	A	0.039	0.005	0.730	1.59E-16	eGFRcrea	0.005	3.83E-04	0.720	5.22E-39
rs17216707	T	C	0.054	0.005	0.800	3.00E-24	eGFRcrea	-0.005	4.51E-04	0.800	3.69E-31
rs17479566	T	C	0.031	0.005	0.221	2.01E-09	eGFRcrea	0.003	4.28E-04	0.220	4.22E-11
rs9925837	G	A	0.035	0.006	0.130	5.11E-09	eGFRcrea	0.003	4.83E-04	0.150	5.25E-09
rs11741640	G	A	0.039	0.005	0.730	1.59E-16	eGFRcys	0.005	0.003	0.809	0.046
rs17216707	T	C	0.054	0.005	0.800	3.00E-24	eGFRcys	-0.009	0.003	0.850	1.39E-03
rs17479566	T	C	0.031	0.005	0.221	2.01E-09	eGFRcys	0.008	0.003	0.208	0.006
rs9925837	G	A	0.035	0.006	0.130	5.11E-09	eGFRcys	0.004	0.003	0.177	0.145
rs11741640	G	A	0.039	0.005	0.730	1.59E-16	UACR	-3.63E-04	0.002	0.734	0.872
rs17216707	T	C	0.054	0.005	0.800	3.00E-24	UACR	-0.003	0.003	0.809	0.331
rs17479566	T	C	0.031	0.005	0.221	2.01E-09	UACR	-0.001	0.002	0.220	0.583
rs9925837	G	A	0.035	0.006	0.130	5.11E-09	UACR	-0.003	0.003	0.151	0.322
rs11741640	G	A	0.039	0.005	0.730	1.59E-16	CKD	-0.064	0.010	0.700	2.18E-10
rs17216707	T	C	0.054	0.005	0.800	3.00E-24	CKD	0.045	0.013	0.810	2.92E-04

SNP	EA	OA	FGF23				Outcome	Outcome			
			Beta	SE	EAF	P value		Beta	SE	EAF	P value
rs17479566	T	C	0.031	0.005	0.221	2.01E-09	CKD	-0.037	0.011	0.220	1.09E-03
rs9925837	G	A	0.035	0.006	0.130	5.11E-09	CKD	-0.023	0.013	0.150	0.076
rs17216707	T	C	0.054	0.005	0.800	3.00E-24	Parental attained age	0.004	0.003	0.811	0.220
rs17479566	T	C	0.031	0.005	0.221	2.01E-09	Parental attained age	0.002	0.003	0.219	0.400
rs9925837	G	A	0.035	0.006	0.130	5.11E-09	Parental attained age	3.24E-04	0.003	0.150	0.920
rs11741640	G	A	0.039	0.005	0.730	1.59E-16	Longevity90^{th}	-0.016	0.022	0.730	0.471
rs17216707	T	C	0.054	0.005	0.800	3.00E-24	Longevity90^{th}	0.012	0.026	0.790	0.651
rs17479566	T	C	0.031	0.005	0.221	2.01E-09	Longevity90^{th}	-0.007	0.023	0.230	0.768
rs9925837	G	A	0.035	0.006	0.130	5.11E-09	Longevity90^{th}	0.031	0.027	0.160	0.238

Note: SNP proxies are defined using 1000 Genomes European sample data with minimum r^2 of 0.8 and maximum minor allele frequency of palindromes is 0.3. SNP, Single nucleotide polymorphism; EA, effect allele; OA, other allele; SE, standard error; EAF, effect allele frequency; CVD, cardiovascular diseases; CAD, coronary artery disease; MI, myocardial infarction; HF: heart failure; AF, atrial fibrillation; FG, fasting glucose; HbA_{1c}, glycated hemoglobin; T2DM, type 2 diabetes mellitus; SBP, systolic blood pressure; DBP, diastolic blood pressure; BMI, body mass index; eGFRcrea, estimated glomerular filtration rate based on creatinine; eGFRcys, estimated glomerular filtration rate based on cystatin C; UACR, urinary albumin-to-creatinine ratio; CKD, chronic kidney disease; Longevity90^{th}, longevity (age ≥ 90th percentile).

Supplementary Table 3. Genetic associations of CVDs and T2DM in FinnGen study.

SNP	EA	OA	Outcome	Sample size	Beta	SE	EAF	P value	F-statistics	Proxy SNP	Proxy SNP chr:position
rs11741640	G	A	Major coronary heart disease	96,499 (N case = 7,123, N control = 89,376)	-0.031	0.022	0.725	0.173	60.8	rs4075958	5:176784512
rs17216707	T	C	Major coronary heart disease	96,499 (N case = 7,123, N control = 89,376)	0.022	0.025	0.791	0.368	117.5		
rs9925837	G	A	Major coronary heart disease	96,499 (N case = 7,123, N control = 89,376)	-0.032	0.027	0.161	0.245	34.0		
rs11741640	G	A	T2DM	95,030 (N case = 12,375, N control = 82,655)	-0.017	0.017	0.725	0.340	60.8	rs4075958	5:176784512
rs17216707	T	C	T2DM	95,030 (N case = 12,375, N control = 82,655)	-0.050	0.019	0.791	0.009	117.5		
rs9925837	G	A	T2DM	95,030 (N case = 12,375, N control = 82,655)	0.051	0.021	0.161	0.015	34.0		

Data source: FinnGen study (14-January-2020 release)

Note: LD proxies are defined using web-based LDlink (website: https://ldlink.nci.nih.gov/?tab=home) within population of Finnish in Finland with r^2 of 1 and D' of 1.

SNP, Single nucleotide polymorphism; T2DM, type 2 diabetes mellitus; EA, effect allele; OA, other allele; SE, standard error; EAF, effect allele frequency.

Supplementary Table 4. The associations of genetically predicted FGF23 and CAD and T2DM in FinnGen study using Mendelian randomization.

Outcome	Exposure	Method	Number of SNPs	OR	(95% CI)	P value	P value of MR-Egger intercept	P value of Cochran's Q
Major coronary heart disease	FGF23	IVW	3	0.82	(0.35, 1.93)	0.645		0.163
Major coronary heart disease	FGF23	MR-Egger	3	21.24	(0.67, 676.71)	0.334	0.312	
Major coronary heart disease	FGF23	WM	3	0.74	(0.35, 1.57)	0.436		
T2DM	FGF23	IVW	3	0.71	(0.22, 2.33)	0.572		0.003*
T2DM	FGF23	MR-Egger	3	0.02	(0.67E-05, 2.62)	0.357	0.377	
T2DM	FGF23	WM	3	0.53	(0.30, 0.95)	0.033		

SNP, Single nucleotide polymorphism; IVW, inverse-variance weighted; WM, weighted median; OR, odds ratio; CI, confidence interval; T2DM, type 2 diabetes mellitus. * Significance at level of 0.05.

Supplementary Table 5. Genetic association of rs2769071 in *ABO* gene with major CVD, their risk factors, kidney function and longevity.

SNP	EA	OA	Exposure				Outcome	Outcome			
			Beta	SE	EAF	P value		Beta	SE	EAF	P value
rs2769071	G	A	0.037	0.005	0.370	6.08E-17	CAD	0.044	0.009	0.377	3.28E-06
rs2769071	G	A	0.037	0.005	0.370	6.08E-17	MI	0.078	0.011	0.369	1.91E-13
rs2769071	G	A	0.037	0.005	0.370	6.08E-17	HF	0.048	0.010	0.353	3.82E-07
rs2769071	G	A	0.037	0.005	0.370	6.08E-17	AF	0.010	0.008	NA	0.200
rs2769071	G	A	0.037	0.005	0.370	6.08E-17	FG	-3.13E-04	0.004	NA	0.937
rs2769071	G	A	0.037	0.005	0.370	6.08E-17	T2DM	0.034	0.011	0.300	0.002
rs2769071	G	A	0.037	0.005	0.370	6.08E-17	SBP	-0.002	0.002	0.320	0.300
rs2769071	G	A	0.037	0.005	0.370	6.08E-17	DBP	-0.016	0.002	0.320	4.10E-13
rs2769071	G	A	0.037	0.005	0.370	6.08E-17	BMI	0.002	0.002	0.335	0.220
rs2769071	G	A	0.037	0.005	0.370	6.08E-17	eGFRcrea	0.001	3.89E-04	0.350	2.53E-03
rs2769071	G	A	0.037	0.005	0.370	6.08E-17	eGFRcys	-0.005	0.002	0.375	0.028
rs2769071	G	A	0.037	0.005	0.370	6.08E-17	UACR	-0.001	0.002	0.326	0.543
rs2769071	G	A	0.037	0.005	0.370	6.08E-17	CKD	-0.012	0.011	0.360	0.299
rs2769071	G	A	0.037	0.005	0.370	6.08E-17	Longevity90th	-0.028	0.020	0.350	0.173

Note: SNP proxies are defined using 1000 Genomes European sample data with minimum r^2 of 0.8 and maximum minor allele frequency of palindromes is 0.3. SNP, Single nucleotide polymorphism; EA, effect allele; OA, other allele; SE, standard error; EAF, effect allele frequency; CVD, cardiovascular diseases; CAD, coronary artery disease; MI, myocardial infarction; HF: heart failure; AF, atrial fibrillation; FG, fasting glucose; T2DM, type 2 diabetes mellitus; SBP, systolic blood pressure; DBP, diastolic blood pressure; BMI, body mass index; eGFRcrea, estimated glomerular filtration rate based on creatinine; eGFRcys, estimated glomerular filtration rate based on cystatin C; UACR, urinary albumin-to-creatinine ratio; CKD, chronic kidney disease; Longevity90th, longevity (age $\geq$ 90th percentile).

Supplementary Table 6. Participant overlap between the FGF23 genome wide association studies (GWAS) and the outcome GWAS.

Outcomes	Cohorts of FGF23 GWAS (N = 16,624)							Sample overlap[#]
	ARIC, US (N = 8,594)	CHS, US (N = 1,988)	US (N = 1,128)	MrOS GBG,	MESA, US (N = 2,163)	MrOS Malmo, Sweden (N = 894)	OPRA, Sweden (N = 920)	
CAD (N = 184,305)	✓							4.7%
MI (N = 166,065)	✓							5.2%
HF (N =	✓	✓						
AF (N = 537,409)	✓	✓						2.0%
FG (N = 140,595)	✓	✓						7.5%
HbA_{1c} (N = 123,665)	✓				✓			8.7%
								0
								0
								0
BMI (N = 681,275)								0
eGFRcrea (N = 567,460)	✓	✓			✓			
	✓				✓			44.7%
UACR (N = 547,361)	✓	✓			✓			2.3%
CKD (N = 480,698)	✓	✓			✓			
Parental attained age (N = 389,166)								0
Longevity 90th (N = 36,745)		✓						5.4%

ARIC, Atherosclerosis Risk in Communities Study; CHS, Cardiovascular Health Study; Indiana, Indiana Sisters Study; MrOS GBG, Osteoporotic Fractures in Men Study–Goteborg; MESA, Multi-Ethnic Study of Atherosclerosis; OPRA, Osteoporosis Prospective Risk Assessment Study; US, United States; CVD, cardiovascular diseases; CAD, coronary artery disease; MI, myocardial infarction; HF: heart failure; AF, atrial fibrillation; FG, fasting glucose; HbA_{1c}, glycated hemoglobin; T2DM, type 2 diabetes mellitus; SBP, systolic blood pressure; DBP, diastolic blood pressure; BMI, body mass index; eGFRcrea, estimated glomerular filtration rate based on creatinine; eGFRcys, estimated glomerular filtration rate based on cystatin C; UACR, urinary albumin-to-creatinine ratio; CKD, chronic kidney disease; Longevity90th, longevity (age ≥ 90th percentile).

[#] Assume there is 100% sample overlap if the outcome GWAS is contributed from the sample cohort.

✓ Presence of Sample overlap

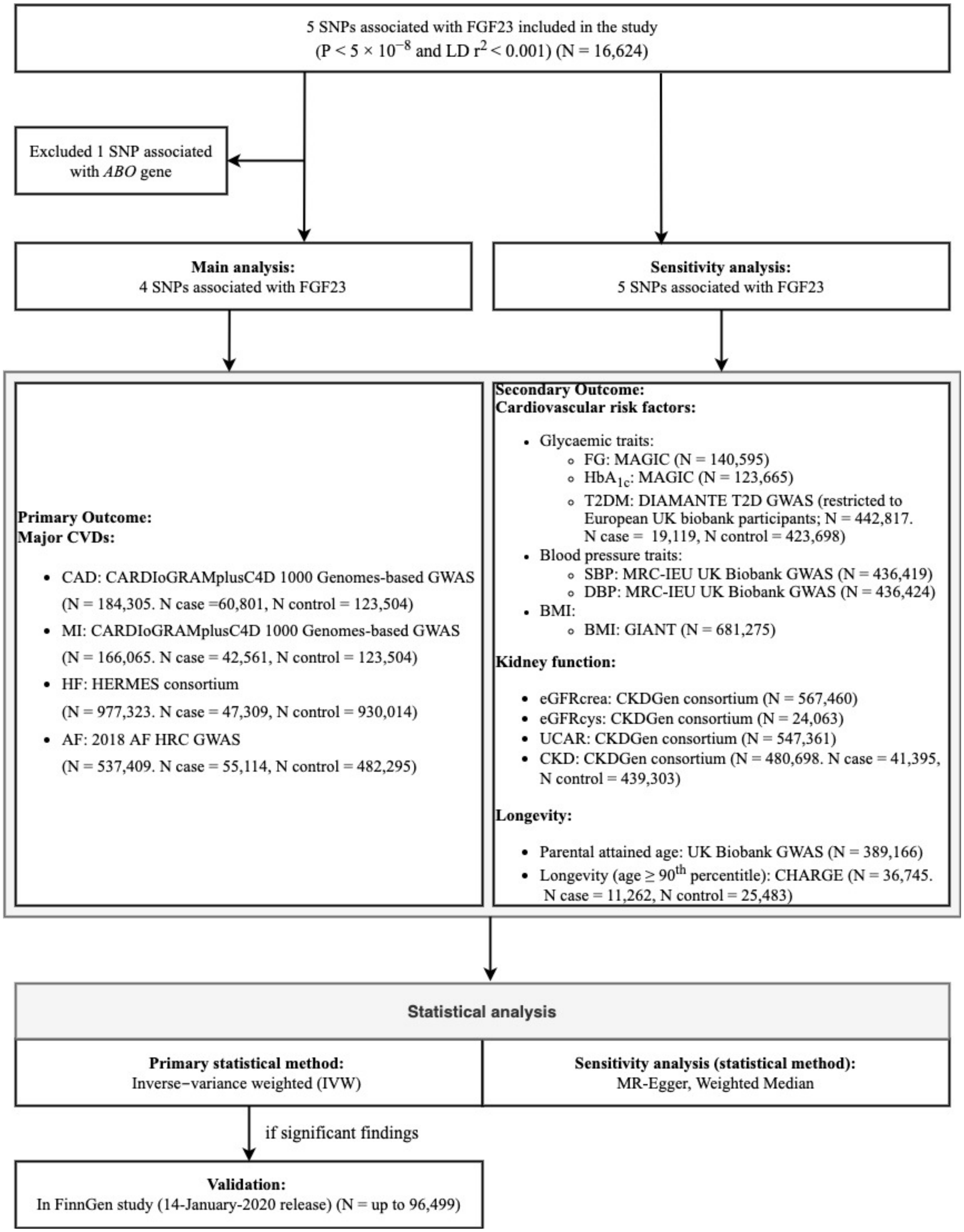

Supplementary Figure 1. Study design of this Mendelian randomization study of genetically predicted FGF23 and cardiovascular diseases, their risk factors, kidney function and longevity. SNP, single nucleotide polymorphism; LD, linkage disequilibrium; CARDIoGRAMplusC4D, Coronary ARtery DIsease Genome wide Replication and Meta-analysis (CARDIoGRAM) plus The Coronary Artery Disease (C4D) Genetics consortium; GWAS, Genome-wide association

study; HERMES, The Heart Failure Molecular Epidemiology for Therapeutic Targets; HRC, Haplotype Reference Consortium; MAGIC, Meta-Analyses of Glucose and Insulin-related traits Consortium; DIAMANTE, DIAbetes Meta-ANalysis of Trans-Ethnic association studies; MRC-IEU, Medical Research Council-Integrative Epidemiology Unit; GIANT, Genetic Investigation of ANthropometric Traits; CKDGen, Chronic Kidney Disease Genetics; CHARGE, Cohorts for Health and Aging in genomic Epidemiology; CVD, cardiovascular diseases; CAD, coronary artery disease; MI, myocardial infarction; HF: heart failure; AF, atrial fibrillation; FG, fasting glucose; HbA1c, glycated hemoglobin; T2DM, type 2 diabetes mellitus; SBP, systolic blood pressure; DBP, diastolic blood pressure; BMI, body mass index; eGFRcrea, estimated glomerular filtration rate based on creatinine; eGFRcys, estimated glomerular filtration rate based on cystatin C; UACR, urinary albumin-to-creatinine ratio; CKD, chronic kidney disease.

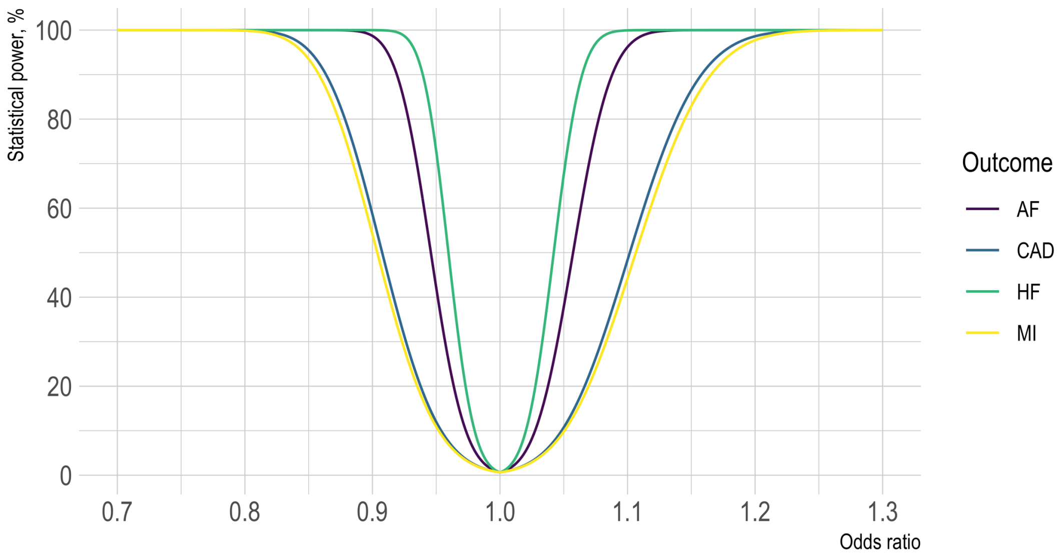

Supplementary Figure 2. Power estimates for this Mendelian randomization of FGF23 in major cardiovascular outcomes. Blue line, power estimate for coronary artery disease (CAD) outcome with a sample size of 184,305 (No. of case: 60,801); Yellow line, power estimate for myocardial infarction (MI) outcome with a sample size of 166,065 (No. of case: 42,561); Green line: power estimate for heart failure (HF) outcome with a sample size of 977,323 (No. of case: 47,309); Purple line: power estimate for atrial fibrillation (AF) with a sample size of 537,409 (No. of case: 55,114). The statistical power was calculated with a significance level of 0.0125 and 1.5% of variance of FGF23 explained by 4 SNPs. This Mendelian randomization analyses reach 80% statistical power for Odds ratio (OR) of ≤ 0.878 or ≥ 1.139 for CAD, OR of ≤ 0.873 or ≥ 1.145 for MI, OR of ≤ 0.946 or ≥ 1.057 for HF, and OR of ≤ 0.928 or ≥1.078 for AF.

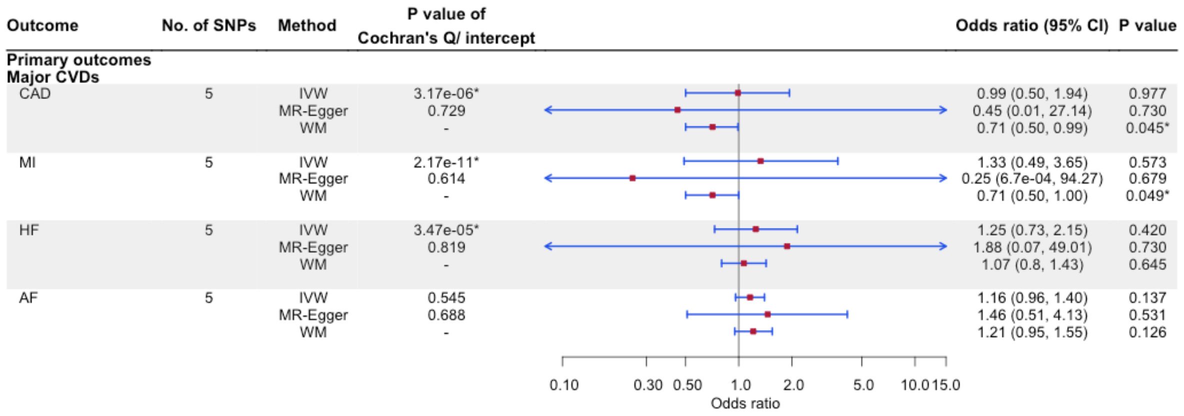

Supplementary Figure 3. The associations of genetically predicted FGF23 and major cardiovascular diseases using Mendelian randomization, including the rs2769071 in *ABO* gene. No. of SNPs, number of single nucleotide polymorphisms; IVW, inverse-variance weighted; WM, weighted median; CVD, cardiovascular disease; CAD, coronary artery disease; MI, myocardial infarction; HF, heart failure; AF, atrial fibrillation. * P value < 0.05.

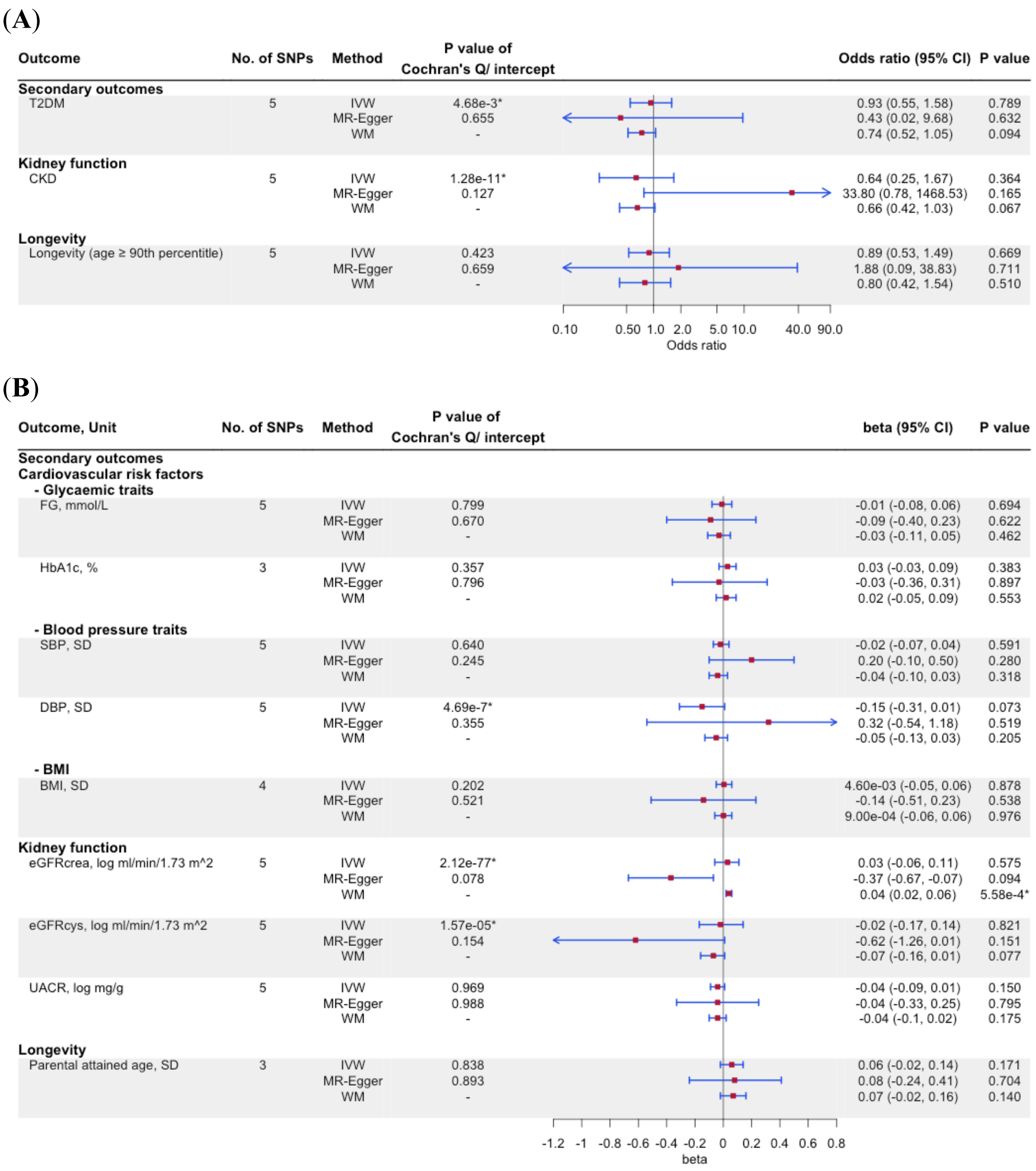

Supplementary Figure 4. The associations of genetically predicted FGF23 on cardiovascular risk factors, kidney function and longevity using Mendelian randomization, including the rs2769071 in *ABO* gene. (**A**) Type 2 diabetes mellitus, chronic kidney disease and longevity; (**B**) Glycaemic traits, blood pressure traits, BMI, kidney function and longevity. No. of SNPs, number of single nucleotide polymorphisms; IVW, inverse-variance weighted; WM, weighted median; T2DM, type 2 diabetes mellitus; FG, fasting glucose; HbA_{1c}, glycated hemoglobin; SBP, systolic blood pressure; DBP, diastolic blood pressure; BMI, body mass index; eGFRcrea, estimated glomerular filtration rate from creatinine; eGFRcys, estimated glomerular filtration rate from cystatin C; UACR, urinary albumin-to-creatinine ratio; CKD, chronic kidney disease. * P value < 0.05.
